# Solitary Melanoma Lesion in a Pulmonary Nodule: A Case Report

**DOI:** 10.7759/cureus.85472

**Published:** 2025-06-06

**Authors:** Gabrielle Aluisio, Grace Ralston, Gene Saylors

**Affiliations:** 1 Medicine, Edward Via College of Osteopathic Medicine, Spartanburg, USA; 2 Oncology, Bon Secours St. Francis Hospital, Charleston, USA

**Keywords:** melanoma, metastatic melanoma, nivolumab-relatlimab, primary malignant melanoma of the lung (pmml), solitary pulmonary nodule

## Abstract

Most melanomas in the respiratory system present as metastatic tumors, with primary lung melanoma nodules being incredibly rare. Melanoma metastasis to the lung is often the first clinically apparent site of visceral metastasis. Any pulmonary lesion confirmed to be melanoma must be assumed to represent metastatic disease until proven otherwise, since it is much more common than a primary lesion. This case describes a 63-year-old female with an incidental 12 x 11 mm nodule found on chest computed tomography (CT) in the upper lobe of the left lung. This nodule appeared to be new, as evidenced by a previous cardiac CT performed one year prior that should have had the nodule in the field of view. Positron emission tomography/CT (PET/CT) demonstrated a fluorodeoxyglucose (FDG)-avid solid left upper lobe nodule with no evidence of regional lymph node involvement or distant metastatic disease, concluding the nodule was most consistent with a primary pulmonary neoplasm. Magnetic resonance imaging (MRI) of the brain confirmed no evidence of brain metastatic disease. CT-guided fine-needle biopsy was performed, with histology consistent with malignant melanoma. The tumor board agreed that, despite the lack of evidence of melanoma on skin exam, fundus exam, or imaging of distant sites, the patient’s melanoma should be treated as metastatic melanoma of unknown primary site (cTx, cN0, cM1b, stage IV). Treatment with nivolumab-relatlimab 280 mg intravenously every 28 days was initiated, with a significant treatment response within two infusions. This case report discusses the diagnosis and approach to a solitary lung melanoma suspicious for primary pulmonary malignant melanoma, with no clear evidence of metastasis, with current therapeutic guidelines for metastatic melanoma cases.

## Introduction

Melanoma is an aggressive malignancy derived from the transformation of melanocytes that are embryologically derived from neural crest cells [1]. Melanoma most commonly arises on the skin and can spread locally, regionally, and distantly; however, melanoma can develop in other locations where neural crest cells migrate [1]. The American Cancer Society estimated that in 2025, about 104,960 new cutaneous melanoma cases will be diagnosed, and 8,430 people are expected to die of melanoma [2]. Of the 359,822 melanoma of the skin cases between 2012 and 2021, 77.8% of cases remained localized, while 9.3% spread regionally, 4.7% spread distantly, and 8.2% remained unstaged [3]. The one-year survival rates in melanoma patients with clinically apparent metastasis to one, two, or three different visceral sites are 36%, 13%, and 1%, respectively [4,5].

Metastasis to the lung is often the first clinically apparent site of visceral melanoma metastasis [5]. Other common sites of metastasis include skin, brain, liver, bone, and intestine [4,5]. Most melanomas in the respiratory system present as metastatic tumors, with primary lung melanoma comprising 0.01% of all primary lung tumors [6]. Metastatic melanomas involving the lung account for 5% of all malignant metastases [7]. Melanoma can originate in the lungs as a primary tumor from melanocytes derived from the neural crest during development [5]. Any pulmonary lesion confirmed to be melanoma must be assumed to represent metastatic disease until proven otherwise, since it is much more common than a primary lesion. The five-year survival of stage 4 melanoma metastatic to the lung is 7-9% [4,8]. Although there is little data regarding primary melanoma of the lung, it is estimated that the five-year survival is at least 10% [8]. Approximately 5-10% of metastatic melanomas have a primary melanoma of unknown origin [9]. In 2017, there were 41 patients with cases of pulmonary malignant melanoma in the English literature [9].

## Case presentation

**Figure 1:**
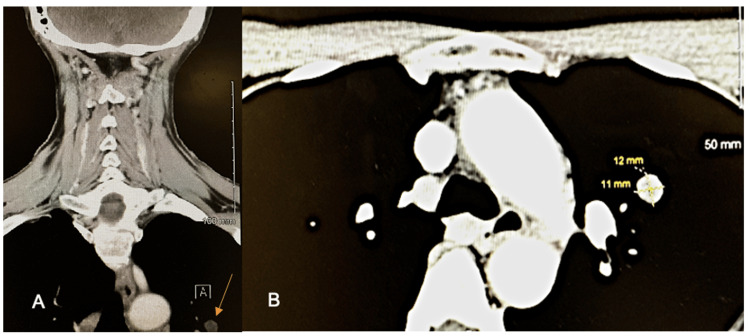
CT soft tissue neck showing an incidental 12 x 11 mm nodule in upper lobe of the left lung in (A) coronal plane (orange arrow) and (B) transverse plane.

A 63-year-old female presented to her primary care physician with a chief complaint of chronic right-sided sialadenitis. Her past medical history included hyperlipidemia, coronary artery disease, osteoarthritis, and non-metastatic cutaneous squamous cell carcinoma treated with local wide excision with negative surgical margins. She reports night sweats, fatigue, shortness of breath, mucus-producing cough, and hip and leg pain. A CT soft tissue neck demonstrated an incidental 12 x 11 mm nodule in the upper lobe of the left lung (Figure 1). This nodule appeared to be new, as evidenced by the absence of the nodule on a previous cardiac CT performed one year prior. The patient was referred to CT surgery and oncology for further workup and management.

**Figure 2:**
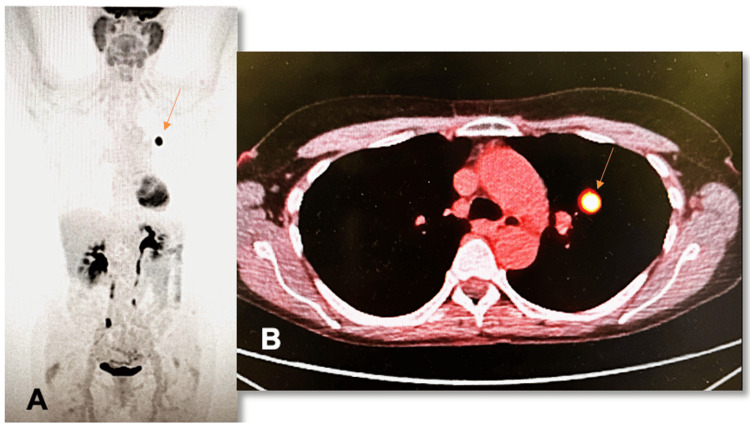
PET/CT scan showing an FDG-avid solid left upper lobe nodule with no evidence of regional lymph node involvement or distant metastatic disease (orange arrow) in (A) coronal and (B) transverse plane. PET/CT: positron emission tomography/computed tomography; FDG: fluorodeoxyglucose

Given the patient’s absent smoking history, high fluorodeoxyglucose (FDG) standardized uptake value (SUV) of 12, and the nodule’s round, smooth borders on imaging, a biopsy was recommended. CT-guided fine-needle biopsy demonstrated cells positive for SOX10, MART1, and S100, and negative for cytokeratin, consistent with malignant melanoma. Caris testing (Caris Life Sciences, Irving, USA) demonstrated elevated tumor mutational burden (TMB), BRAF wild-type, and mutations in NRAS, NF1, RAC1, and TERT promoter genes. A whole-body PET/CT demonstrated an FDG-avid, 1.4 cm solid left upper lobe nodule with no evidence of regional lymph node involvement or distant metastatic disease, suggestive of a primary pulmonary neoplasm (Figure 2). There was no evidence of metastatic disease or additional hypermetabolic disease on brain magnetic resonance imaging (MRI). Extensive dermatological and ocular examination revealed no identifiable lesions.

**Figure 3:**
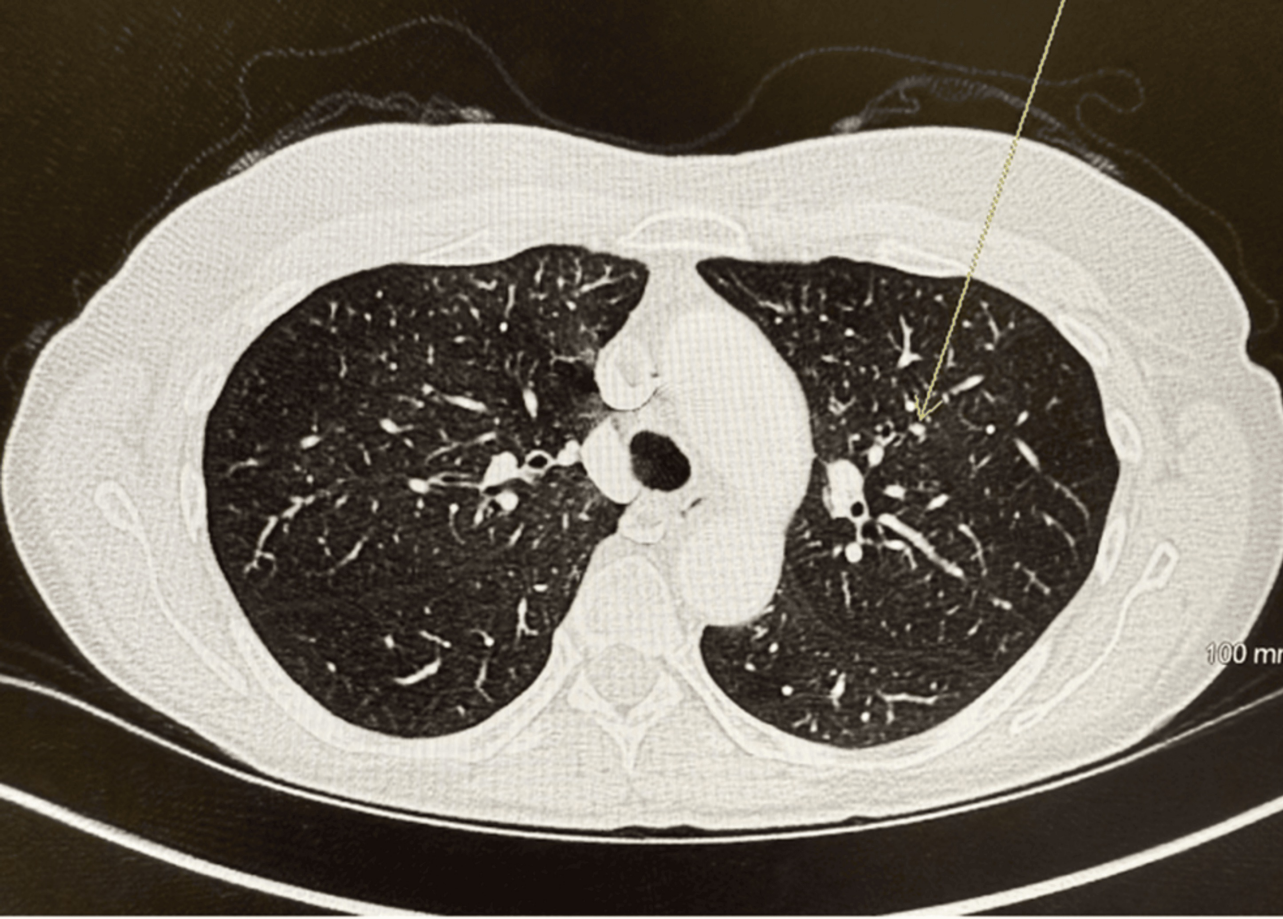
Pulmonary nodule after two treatments with nivolumab plus relatlimab (yellow arrow).

An interdisciplinary tumor board agreed that the melanoma should be treated as metastatic melanoma of unknown primary site (cTx cN0, cM1b, Stage IV). Treatment with nivolumab-relatlimab 280 IV every 28 days was initiated. The patient received two treatments with nivolumab-relatlimab therapy and reported none of the common side effects, such as fatigue, low-grade rash, and generalized musculoskeletal discomfort with focal arthritis. Repeat CT abdomen/pelvis performed eight weeks after initiation of treatment showed markedly decreased size of left upper lobe nodule (Figure 3). The patient received an additional nivolumab-relatlimab infusion. Since that infusion, the patient transferred care to a new city with plans to follow up accordingly.

## Discussion

The pathophysiology of primary pulmonary malignant melanoma is still highly debated. The most widely accepted proposed mechanisms include the proliferation of melanocytes into the larynx and esophagus versus melanocyte migration during embryogenesis [10]. It is also possible that a primary malignant cutaneous melanoma has formed and disappeared after metastasizing in cases where no primary lesion can be identified, which is the most likely etiology in this case [10,11]. Thus, the most important consideration when working up a diagnosis in a case like this is to exclude metastasis as the origin of the disease. Allen and Drash proposed the following criteria to further support the diagnosis of primary malignant melanoma of the lung (PMML): (1) no history that is suggestive of a prior melanoma; (2) no melanoma in any other organ at the time of surgery; (3) tumor is solitary in nature within lung parenchyma; (4) tumor morphology is compatible with that of the primary tumor; (5) no evidence of melanoma elsewhere at autopsy; (6) melanoma cells stain positive for S-100 and HMB-45; (7) evidence of junctional change present; (8) presence of "nesting" of cells beneath the bronchial epithelium; and (9) evidence of melanoma cells invading intact bronchial epithelium [10].

The first-line treatment for PMML is surgical resection [10]. Inoperable tumors historically have been treated with chemotherapy, usually dacarbazine, and immunotherapy with interferon or interleukin-2 is considered [10,11]. First-line treatment for metastatic melanoma includes resection of the primary lesion, if possible, with adjuvant systemic therapy [12]. Preferred systemic immunotherapy regimens for metastatic cases include nivolumab, pembrolizumab, and nivolumab/ipilumab [12]. If the melanoma is BRAF V600E mutation positive, then kinase inhibitors such as dabrafenib/trametinib, vemurafenib/cobimetinib, and encorafenib/binimetinib are preferred [12]. Chemotherapy was considered for this patient's case; however, immunotherapy was more desirable given the promising efficacy rates for primary cutaneous melanomas and metastatic cases. Despite the patient's comorbidities, her health was well-managed with good functional status, which further supported the use of nivolumab, a programmed cell death protein 1 (PD-1) inhibitor, and relatlimab, a lymphocyte-activation gene 3 (LAG-3) inhibitor, over chemotherapy. The patient's regimen choice was supported by Amaria et al., who stated this combination resulted in a 57% pathologic complete response rate and 70% overall pathologic response rate with no grade 3-4 immuned-related adverse events observed [13]. Those with any pathologic response had a one- and two-year recurrence-free survival rate of 100% and 92% compared to 88% and 55% for those without pathologic response [13].

The patient had a dramatic clinical response to two infusions of nivolumab plus relatlimab with no adverse effects. This case contributes to the marginal literature on PMML by demonstrating an alternative approach to treating PMML by following the metastatic melanoma National Comprehensive Cancer Network (NCCN) guidelines [12].

## Conclusions

It is unclear whether this case is reflective of metastatic melanoma with a since-disappeared primary lesion or a PMML. Although metastatic melanoma is much more common, this lesion fell in line more with the proposed criteria to support the diagnosis of PMML. The decision to treat following a metastatic lesion protocol was supported by clinical response and survival rate to the treatment. The patient had a dramatic clinical response to two infusions of nivolumab plus relatlimab with no adverse effects. This case contributes to the marginal literature on PMML by demonstrating an alternative approach to treating PMML by following the metastatic melanoma national treatment guidelines.
